# A 7-Amino Acid Peptide Mimic from Hepatitis C Virus Hypervariable Region 1 Inhibits Mouse Lung Th9 Cell Differentiation by Blocking CD81 Signaling during Allergic Lung Inflammation

**DOI:** 10.1155/2020/4184380

**Published:** 2020-03-20

**Authors:** Wanzhou Zhao, Conghao Tan, Xi Yu, Ruihe Yu, Qibing Mei, Yun Cheng

**Affiliations:** ^1^The Nanjing Han & Zaenker Cancer Institute (NHZCI), OG Pharmaceuticals, Jiangdongbei Road 88, Nanjing, 210036 Jiangsu Province, China; ^2^School of Medicine, Jiangsu University, 301 Xuefu Road, Zhenjiang, 212013 Jiangsu Province, China; ^3^Luzhou New Drug Evaluation and Research Center, Luzhou, 646000 Sichuan Province, China

## Abstract

T helper (Th) cells orchestrate allergic lung inflammation in asthma pathogenesis. Th9 is a novel Th cell subset that mainly produces IL-9, a potent proinflammatory cytokine in asthma. A 7-amino acid peptide (7P) of the hypervariable region 1 (HVR1) of hepatitis C virus has been identified as an important regulator in the type 2 cytokine (IL-4, IL-5, and IL-13) immune response. However, it is unknown whether 7P regulates Th9 cell differentiation during ovalbumin- (OVA-) induced allergic lung inflammation. To address this, we studied wild-type mice treated with 7P and a control peptide in an *in vivo* mouse model of OVA-induced allergic inflammation and an *in vitro* cell model of Th9 differentiation, using flow cytometry, cytokine assays, and quantitative PCR. The binding of 7P to CD81 on naïve CD4^+^ T cells during lung Th9 differentiation was determined using CD81 overexpression and siRNA knockdown analyses. Administration of 7P significantly reduced Th9 cell differentiation after OVA sensitization and exposure. 7P also inhibited Th9 cell differentiation from naïve and memory CD4^+^ T cells *in vitro*. Furthermore, 7P inhibited the differentiation of human Th9 cells with high CD81 expression from naïve CD4^+^ T cells by blocking CD81 signaling. CD81 siRNA significantly reduced Th9 cell differentiation from naïve CD4^+^ T cells *in vitro*. Interestingly, CD81 overexpression in human naïve CD4^+^ T cells also enhanced Th9 development *in vitro*. These data indicate that 7P may be a good candidate for reducing IL-9 production in asthma.

## 1. Introduction

Asthma is a chronic airway disease characterized by inflammation, airway hyperreactivity (AHR), and reversible airway obstruction [1]. Complicated innate and adaptive immune responses play significant roles in airway inflammation. Allergen-specific T helper (Th) 2 cells produce key cytokines (IL-4, IL-5, IL-9, and IL-13) for this process [2]. Th9 cells were first identified as a Th2 subpopulation that produced exceptionally large quantities of cytokine IL-9. IL-9 has various effects on numerous hematopoietic cells, which are central to asthma pathogenesis. IL-9 enhances T and mast cell (MC) proliferation and differentiation and promotes IgE production by B cells [3]. The association of IL-9 with susceptibility to developing AHR has been confirmed via human and murine studies [4]. Furthermore, IL-9 elicits eosinophilic and lymphocyte inflammation, mucus production, AHR, and subepithelial collagen deposition in the lungs of mice. The use of neutralizing IL-9 antibodies has been shown to reduce inflammation and AHR. Studies using IL-9-deficient mice demonstrated the redundant role of this cytokine in a similar model of asthma [5]. Mapping studies in mice and humans have shown that IL-9 is an asthma-related gene [6], and anti-IL-9 antibodies have been trialed to treat asthma patients in a clinical setting [7].

The hypervariable region 1 (HVR1) of the hepatitis C virus (HCV) has been identified as an important regulatory factor in the type 2 cytokine (IL-4, IL-5, and IL-13) immune response [8–11]. Therefore, some mimic peptides derived from HVR1 of HCV were investigated for their roles in the immune response. A designated 7-amino acid peptide (7P; GQTYTSG) was identified by its ability to induce an interesting cytokine profile. The expression levels of IFN-*γ*, IL-10, and TNF-*α* in PBMC of HCV patients could be regulated by the 7P stimulation [12, 13]. However, it was unknown whether 7P could regulate lung Th9 differentiation, inflammatory responses, and AHR during ovalbumin (OVA)-induced allergic lung inflammation. It has been reported that HVR1 of HCV interacted with hepatocytes, CD4^+^ T cells, and CD8^+^ T cells through surface CD81 molecules [14]. CD81 is a widely expressed cell-surface protein and is associated with a variety of biological responses in the immune system [15]. More studies have confirmed that CD81 is associated with T and B cell differentiation and expression [16, 17]. Furthermore, CD81 is related to MHC class II molecules, integrins, and other tetraspanins [18]. Studies also suggest that CD81 is involved in cell motility, adhesion, proliferation, and differentiation. CD81-deficient (CD81^−/−^) mice and chimeric mice, in which only B cells lack CD81, have been demonstrated to have impaired humoral immune responses to protein antigens (Ags) [19] and Th2 responses [20]. Furthermore, allergen-induced AHR is diminished in CD81^−/−^ mice through the regulation of Th2 cell differentiation and function [21]. These studies have shown that CD81 is involved in allergen-induced AHR via Th2 regulation. It is still unknown whether CD81 and its ligand are also involved in the regulation of Th9 cells during allergic lung inflammation.

Th9 cells develop *in vitro* in the presence of IL-4 and transforming growth factor- (TGF-) *β* and secrete high levels of IL-9, IL-10, CCL17, and CCL22 [22]. However, the transcription factors that regulate IL-9 are not well defined. The transcription factors IRF4 and PU.1 are required for IL-9-secreting T cell development [23]. PU.1 binds directly to the IL-9 promoter and is required for the development of allergic inflammation [24]. Although IRF4 binds to the IL-9 promoter, it also induces GATA3 expression during Th2 differentiation [25]. This function may impact Th9 development.

7P is a mimic peptide derived from the HVR1 of HCV, which has been determined to regulate type 2 cytokines such as IL-4, IL-5, IL-9, and IL-13. Furthermore, IL-9 is an important factor in asthma pathogenesis [13, 26, 27], and anti-IL-9 antibody has been shown to treat asthma in phase II clinical trials [7]. In this study, we therefore investigated the effect of 7P in regulating lung Th9 cell differentiation during allergic lung inflammation *in vivo* and *in vitro*. We compared lung Th9 cell differentiation in 7P and vehicle minipump-treated mice during OVA-induced allergic lung inflammation *in vivo*.

## 2. Materials and Methods

### 2.1. Animals

Male 6–10-week-old C57BL6J mice (50 mice) were purchased from Nanjing Model Animal Research Center (MARC). All animals were provided with sterilized food and water. The study was approved by the Animal Care and Use Committee of the Nanjing Han & Zaenker Cancer Institute (NHZCI) and performed strictly according to the Guide for the Care and Use of Laboratory Animals.

### 2.2. 7P and Sham-7P Peptides

HVR1 mimic peptide 7P (GQTYTSG) and sham-7P (RKNHVGL) were synthesized using solid-phase multipin technology. The sequences and purity (>98%) of 7P and sham-7P were confirmed by HPLC and MS/MS analysis. Amino acid sequences of sham-7P were randomly selected.

### 2.3. OVA-Induced Allergic Airway Inflammation Model

Mice were immunized with OVA (20 *μ*g emulsified in 0.2 mL aluminum hydroxide adjuvant) or vehicle (adjuvant only) by intraperitoneal injection on days 0 and 1. Starting on day 14, mice were exposed to 1% OVA or saline via nebulizer for 30 min per day for four consecutive days. Forty-eight hours after the last exposure, mice were euthanized, and tissues were collected.

### 2.4. Bronchoalveolar Lavage Fluid (BALF) Collection and Isolation of CD4^+^ T Cells

BALF was collected in 2 mL phosphate-buffered saline (PBS). Naïve or allergic CD4^+^ T cells were isolated using kits from Miltenyi Biotec. Cell sorting was used to obtain naïve CD4^+^/CD25^−^/CD44^+low^/CD62L^+^ T cells at >99% purity. Cell cultures were maintained in RPMI 1640 with 10% fetal bovine serum (FBS). Th9 differentiation was induced as described [22].

### 2.5. Dendritic Cell (DC) Isolation

Mouse spleens were placed into single-cell suspensions with a 2 mL syringe. Spleen DCs were enriched from spleen single-cell suspension with an EasySep™ Mouse Pan-DC Enrichment Kit (STEMCELL, Cat: #19763). CD11c^+^ DCs were further sorted with anti-mouse CD11c-PE antibodies from the enriched lung DCs by BD FACSAria II.

### 2.6. DC-T Cell Coculture

DC-T cell cocultures were conducted in 24-well flat-bottom culture plates. Briefly, the purified lung DCs were placed into a 24-well plate and treated with lipopolysaccharides (LPS) from Escherichia coli (10 ng/mL) overnight. The cultural supernatant was removed, and cultures were extensively washed and resuspended in RPMI/10% fetal calf serum (FCS). Naïve CD4^+^ T cells (1 × 10^6^) were then cocultured with anti-CD3 (2 *μ*g/mL), anti-CD28 (1 *μ*g/mL), anti-IFN-*γ* (10 *μ*g/mL), IL-4 (20 ng/mL), TGF-*β* (2 ng/mL), and DCs for 5 days in 1 mL complete culture medium at a 10 : 1 T : DC cell ratio. Finally, each cell sample was collected, and quantitative polymerase chain reaction (qPCR) was conducted to compare *Il9*, *Il10*, *Pu.1*, and *Irf4* to *Gapdh*.

### 2.7. Implantation of Osmotic Minipumps for Delivery of 7P and Sham-7P

7P and sham-7P were dissolved in a vehicle containing 15% ethanol/sterile saline. These were delivered 1 week prior to OVA exposure via subcutaneously implanted osmotic minipumps (Alzet, Cupertino, CA, model 1004). Minipumps were filled with 100 *μ*L of either 6 *μ*g/mL 7P or sham-7P. With an average release rate of 0.11 *μ*L/h, this provided a final delivery rate of 1.32 *μ*g/h for 7P and sham-7P.

### 2.8. Flow Cytometry Analysis and Intracellular Cytokine Staining

After stimulation of Th9 differentiation from naïve CD4^+^ T cells with TGF-*β* and IL-4, cells were incubated for 4 h with 500 ng/mL 12-O-tetradecanoylphorbol-13-acetate, 500 ng/mL ionomycin (Sigma-Aldrich), and 1 *μ*g/mL brefeldin A (GolgiPlug; BD). Cells were fixed and permeabilized using BD Cytofix/Cytoperm™ solution after surface staining. Phycoerythrin- (PE-) conjugated anti-IL-9 mAb (BioLegend) was used to detect intracellular IL-9 and IL-10 levels. Naïve CD4^+^ T cells and Th9 cells were stained with the conjugated antibodies for 10 min. Cells stained with the appropriate antibodies were analyzed using an LSRII flow cytometer (Becton Dickinson). The data were collected and analyzed using FACSDiva software.

### 2.9. RNA Isolation and Real-Time PCR

Total RNA was isolated using an RNeasy mini kit (Qiagen), and cDNA was synthesized with a High-Capacity cDNA Archive Kit (Applied Biosystems). TaqMan primer/probe sets for mouse GAPDH (Mm99999915_m1), IL-9 (Mm00434305_m1), IL-10 (Mm00439614_m1), and IRF4 (Mm00516431_m1) were purchased from Applied Biosystems. Samples were analyzed using an ABI Prism 7700 Thermocycler (Applied Biosystems), and differential expression was calculated using the *ΔΔ*CT method.

### 2.10. Transient Transfection Assay

Jurkat T cells were transfected in duplicate with the pCDNA3.1-CD81 plasmid or the control pCDNA3.1 plasmid using Lipofectamine 2000 reagent (Invitrogen). Twenty-four hours after transfection, cells were treated with TGF-*β* and IL-4 for 5 days. IL-9, IL-10, and IRF4 levels were quantified by real-time PCR.

### 2.11. siRNA Knockdown of CD81 Receptors

Multiple MISSION siRNAs for each receptor were purchased from Sigma-Aldrich for knockdown analysis (Human CD81: SASI_Hs01_00181702; SASI_Hs01_00181703; SASI_Hs01_001817024). Naïve CD4^+^ T cells freshly isolated from the spleens of wild-type mice were transfected with CD81-specific siRNAs (20 nmol) or control siRNA using Mouse T Cell Nucleofector Solution (Amaxa). Transfected cells were differentiated into Th9 cells and analyzed via FACS on day 5.

### 2.12. Histopathological Staining of Lung Tissue

Lungs were perfusion-fixed, and adjacent sections were stained with hematoxylin and eosin (H&E). Pathological evaluation was performed by a pathologist.

### 2.13. Enzyme-Linked Immunosorbent Assay (ELISA)

BALF samples were also taken to measure IL-5, IL-9, and IL13 production. Briefly, BALF samples were collected in 2 mL PBS. An ELISA kit (eBioscience) was used to observe levels of IL-5, IL-9, and IL-13 according to the manufacturer's instructions.

### 2.14. Statistical Analysis

Data are represented as the mean ± standard error of the mean (SEM). Statistical comparisons were performed by randomized-design two-way analysis of variance (ANOVA), followed by the Newman-Keuls post hoc test for more than two groups or unpaired Student's *t*-test for two groups. Statistical significance was defined as *p* < 0.05.

## 3. Results

### 3.1. 7P Significantly Decreases Lung Th9 Cell Differentiation during Allergic Lung Inflammation *In Vivo*

**Figure 1:**
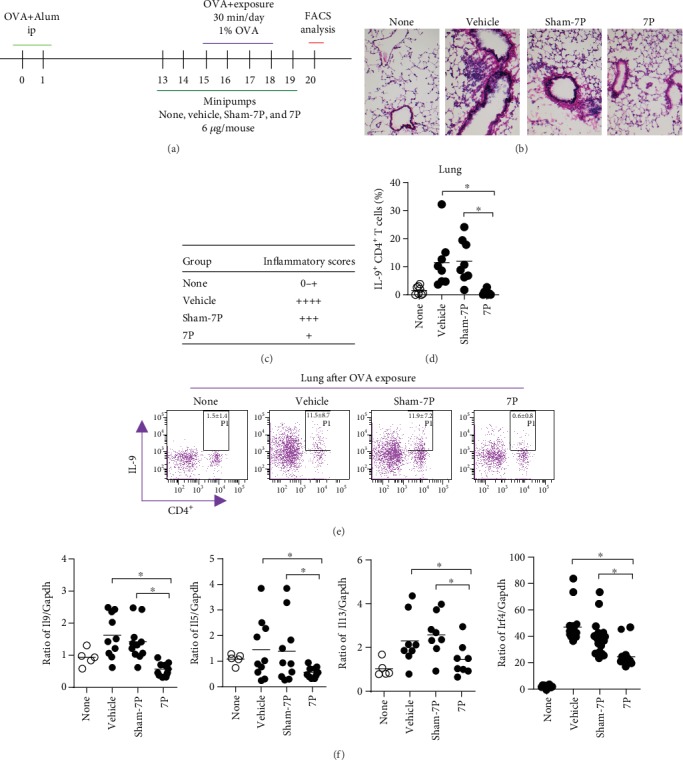
The 7-amino acid peptide (7P) dramatically inhibits Th9 cell differentiation during allergic lung inflammation after ovalbumin (OVA) sensitization and exposure *in vivo*. (a) C57BL6J mice (*n* = 24 each) were sensitized with OVA in adjuvant. Mice were exposed to OVA for inhalation 14–21 days later for 4 consecutive days. Mice were implanted with minipumps from days 13–19; 7P and sham-7P mice were given 6 *μ*g/mouse. (b) Lung tissue sections were stained with hematoxylin and eosin (H&E) and visualized by light microscopy (original magnification 3100). (c) Pathological scoring was done on a traditional 1–4 scale (minimal, mild, moderate, marked) by two pathologists. The inflammatory score was counted by five slides of each normal, 7P-treated group, sham-7P, and vehicle group. (d) The percentages of IL-9^+^CD4^+^ T cells in lung tissue from 7P vs. sham-7P and vehicle minipumped mice were analyzed by flow cytometry 48 h after the last OVA exposure. (e) Flow cytometry scattergrams of IL-9^+^CD4^+^ T cells in the lung. (f) *Il9*, *Il5*, *Il13*, and *Irf4* mRNA in the lung were examined by real-time PCR (^∗^*p* < 0.05, *n* = 12).

Recent progress towards characterization of the proinflammatory IL-9, IL-5, and IL-13 cytokine families of Th9 cells has added a layer of complexity to our understanding of regulating allergic lung inflammation. To determine whether 7P could regulate lung Th9 cells during allergic lung inflammation *in vivo*, we implanted osmotic minipumps containing 7P and vehicle into mice. This allowed us to examine lung Th9 cell differentiation *in vivo* following OVA sensitization/exposure (Figure 1(a)). 7P decreased allergic lung inflammation, as confirmed by H&E staining and information scoring (Figures 1(b) and 1(c)). The percentages of Th9 cells in the lungs of C57BL/c mice in the 7P group were compared with sham-7P and vehicle-treated groups (Figure 1(d)). 7P treatment significantly reduced percentages of Th9 in the lungs (vehicle or sham-7P vs. 7P: 11.48 ± 8.71% and 11.94 ± 7.18% vs. 0.60 ± 0.85%, *n* = 8, *p* < 0.05) compared with sham-7P and vehicle group (Figures 1(d) and 1(e)). Consistent with this result, 7P also significantly suppressed *Il9*, *Il5*, and *Il13* transcripts in lung tissue during allergic lung inflammation (Figure 1(f)). 7P significantly inhibited Th9 cell differentiation in the BALF (vehicle or sham vs. 7P: 14.36 ± 5.42% or 14.86 ± 6.16% vs. 8.28 ± 4.2%, *n* = 8, *p* < 0.05), spleen (vehicle or sham vs. 7P: 7.5 ± 3.60% or 7.1 ± 2.12% vs. 0.3 ± 0.45%, *n* = 8, *p* < 0.05), lymph nodes (vehicle or sham vs. 7P: 1.88 ± 1.34% or 2.08 ± 1.29% vs. 0.49 ± 0.51%, *n* = 8, *p* < 0.05), and blood (vehicle or sham vs. 7P: 3.09 ± 1.84% or 3.21 ± 2.00% vs. 1.43 ± 0.40%, *n* = 8, *p* > 0.05) (Supplementary [Supplementary-material supplementary-material-1]). Furthermore, levels of IL-9, IL-5, and IL-13 protein in BALF were significantly decreased in 7P-treated mice (Supplementary [Supplementary-material supplementary-material-1]). These data suggest that Th9 cell numbers, along with IL-9 and IL-13 cytokine levels, are reduced in the 7P-treated group compared with the sham-7P and vehicle-treated groups during allergic lung inflammation *in vivo*.

### 3.2. 7P Dramatically Suppresses Th9 Differentiation from Both Naïve CD4^+^ T Cells and OVA-Memory CD4^+^ T Cells *In Vitro*

**Figure 2:**
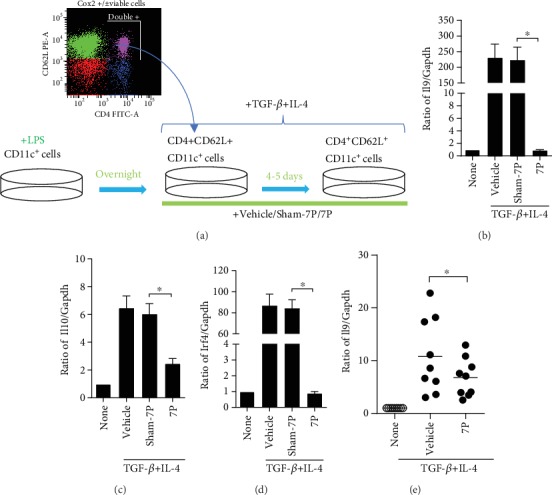
Th9 differentiation from naïve CD4^+^ T cells is inhibited by 7P *in vitro*. (a) Dendritic cells (DC, CD11c^+^ cells) were isolated from the spleen and matured with LPS overnight. Naïve CD4^+^ T cells were then isolated from spleens of C57BL6J mice, and LPS-treated DCs were treated with anti-CD3 (3 *μ*g/mL), anti-CD28 (3 *μ*g/mL), anti-INF-*γ* (3 *μ*g/mL), transforming growth factor- (TGF-) *β* (10 ng/mL), and IL-4 (10 ng/mL) for 4–5 days with 7P, sham-7P, or vehicle. The cells were then restimulated for 4 h with 12-O-tetradecanoylphorbol-13-acetate and ionomycin (500 ng/mL each) in the presence of brefeldin A (1 mg/mL) before real-time PCR analysis. (b–d) Cells treated with TGF-*β* and IL-4 were collected, and prepared RNA was reversed to cDNA. *Il9*, *Il10*, and *Irf4* mRNA were amplified by quantitative polymerase chain reaction (qPCR) after treatment by anti-CD3, anti-CD28, anti-INF-*γ*, TGF-*β*, and IL-4 with 7P, sham-7P, or vehicle. Data were representative of 7–14 independent experiments (*n* = 7–14, ^∗^*p* < 0.05). (e) Human naïve CD4^+^ T cells were isolated from peripheral blood and differentiated into Th9 cells with anti-CD3, anti-CD28, TGF-*β*, and IL-4 plus 7P and vehicle. *Il9* mRNA was also assayed by qPCR (*n* = 5; *p*^∗^ < 0.05).

**Figure 3:**
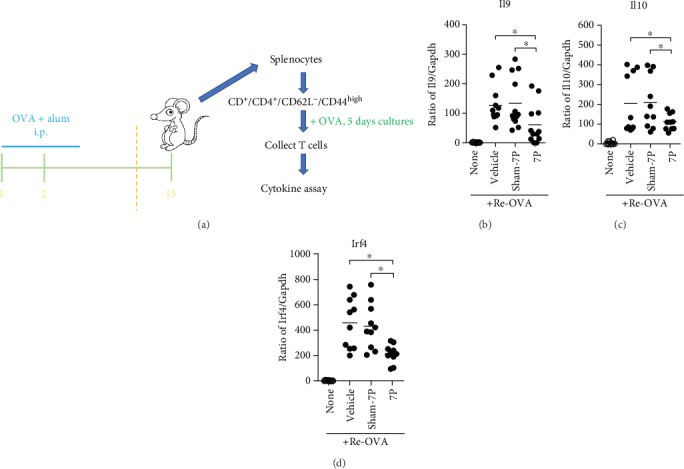
Suppression of Th9 cell differentiation from memory CD4^+^ T cells by 7P. (a) To determine whether 7P could inhibit memory CD4^+^ T cells, 6–8-week-old mice were immunized with OVA and alum (2 *μ*g OVA/mouse) twice on days 1 and 2. After 14 days, CD3^+^CD4^+^CD62L^−^CD44^+high^ CD45RO^+^ T cells (memory CD4^+^ T cells) from the collected spleen were isolated and restimulated by OVA with 7P, sham-7P, or vehicle for 4–5 days. (b–d) RNA was isolated from cells, and *Il9 Il10 lrf4* mRNA was quantified by qPCR. Data were expressed as mean ± standard error of the mean (SEM; *n* = 10 mice/group from 1 to 3 independent experiments). ^∗^*p* < 0.05 compared with sham-7P and vehicle-treated mice.

To examine the mechanism by which 7P inhibits lung Th9 cell differentiation during allergic lung inflammation, we applied an *in vitro* Th9 differentiation method from naïve CD4+ T cells to observe the effect of 7P on suppressing Th9 cell differentiation. Briefly, we treated naïve CD4^+^ T cells isolated from mouse spleens with anti-CD3, anti-CD28, and Th9-inducing cytokines (TGF-*β* and IL-4) to induce Th9 cell differentiation in the presence and absence of 7P, sham-7P, and vehicle (Figure 2(a)). *Il9*, *Il10*, and *Irf4* expressions were investigated to identify Th9 cell differentiation. The results indicated that Th9 cell lineage markers (*Il9*, *IL10*, and *Irf4*) were significantly decreased following treatment of naïve CD4^+^ T cells with TGF-*β*, IL-4, and 7P (Figures 2(b)–2(d)). Human Th9 differentiation from human peripheral naïve CD4^+^ T cells was significantly inhibited by 7P (Figure 2(e)). These data indicate that 7P is a negative regulator of Th9 cell differentiation from naïve CD4^+^ T cells and confirms that IL-9 production is decreased with 7P treatment. Although 7P has been observed to inhibit Th9 cell differentiation from naïve CD4^+^ T cells *in vitro*, it is unknown whether 7P also inhibits Th9 cell differentiation from OVA-memory CD4^+^ T cells. To address this issue, we immunized mice with OVA for 14 days, followed by isolating OVA-memorized CD4^+^ T cells (CD3^+^/CD4^+^/CD62L^−^/CD44^high+^ T cells). We then restimulated them with OVA *in vitro* for 5 days (Figure 3(a)). The OVA-restimulated memory CD4^+^ T cells were collected, and RNA was isolated. *Il9*, *Il10*, and *Irf4* mRNA were quantified by qPCR. The results show that *Il9*, *Il10*, and *Irf4* mRNA were significantly decreased in the 7P-treated group compared with the vehicle and sham-7P groups (Figures 3(b)–3(d)). These data demonstrate that 7P not only suppresses Th9 cell differentiation from naïve CD4^+^ T cells but also inhibits Th9 cell differentiation from memory CD4^+^ T cells.

### 3.3. Th9 Cells Have High CD81 Expression and Can Bind 7P

**Figure 4:**
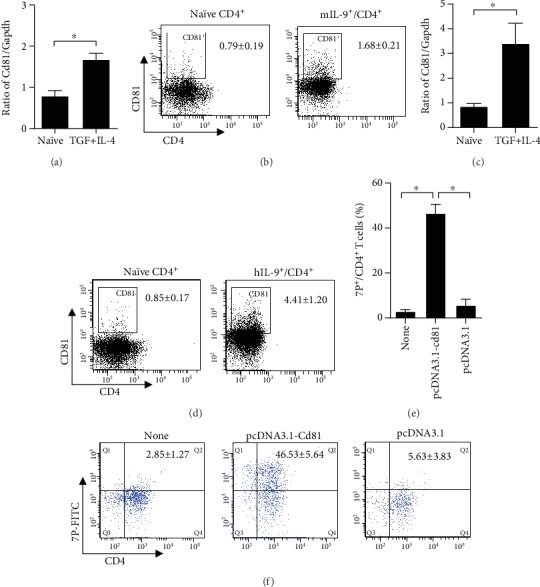
CD81 expression during mouse and human Th9 cell differentiation. Naïve CD4^+^ T cells and 7P bind to CD81. (a–d) Mouse and human naïve CD4^+^ T cells isolated from mouse spleen and human peripheral blood were stimulated with anti-CD3 (3 *μ*g/mL), anti-CD28 (3 *μ*g/mL), and anti-INF-*γ* (3 *μ*g/mL) with TGF-*β* (10 ng/mL) and IL-4 (10 or 20 ng/mL) for 5 days to induce (a) mouse and (c) human Th9 cell differentiation. Cells were analyzed for CD81 expression by qPCR (*n* = 6, ^∗^*p* < 0.05). (b) Mouse and (d) human CD81 were examined by flow cytometry. (e, f) Naïve CD4^+^ T cells transfected with pcDNA3.1-CD81 and purified by FACS. Cells were stimulated with anti-CD3, anti-CD28, anti–INF-*γ*, TGF-*β*, and IL-4 (10 and 20 ng/mL) for 5 days, then stimulated for 4 h with 12-O-tetradecanoylphorbol-13-acetate and ionomycin (500 ng/mL each) in the presence of brefeldin A (1 *μ*g/mL). Intracellular staining was performed with anti-CD4, IL-9, and CD81. CD81 expression was analyzed by flow cytometry with 7P-FITC in the gated CD4^+^IL-9^+^ cell population. The percentage of 7P-positive Th9 cells was examined by FACS. Results were representative of three independent experiments (*n* = 3, ^∗^*p* < 0.05).

It is established that CD81 is expressed in T cells and regulates the differentiation and function of Th1 and Th2 cells [28]. However, it was unknown whether Th9 cells express CD81. Previous studies have demonstrated that CD81 signaling plays an important role in promoting Th2 cells in asthma [29]. Therefore, blocking CD81 signaling may inhibit Th cell differentiation. To define the mechanism by which 7P inhibits Th9 cell differentiation, we firstly examined whether Th9 cells express CD81 during mouse and human Th9 cell differentiation. This was determined *in vitro* by qPCR and flow cytometry. The results indicated that levels of CD81 mRNA (Figure 4(a)) and protein expression (Figure 4(b)) by mouse Th9 cells were significantly increased during Th9 cell differentiation from naïve CD4^+^ T cells compared with the vehicle. Consistent with CD81 expression observed in mouse Th9 cells, expression levels of CD81 mRNA and protein by human Th9 cells were also increased, indicating that human Th9 cells had increased CD81 expression (Figures 4(c) and 4(d)). Secondly, to examine whether 7P binds to CD81, we incubated 7P conjugated with FITC (7P-FITC) with CD4^+^ T cells (Jurkat cells) transfected with pcDNA3.1-CD81 or pcDNA3.1 vector plasmids. High CD81 expression was confirmed by flow cytometry in CD4^+^ T cells transfected with pcDNA3.1-CD81 (Supplementary [Supplementary-material supplementary-material-1]). The results indicated that the CD4^+^ T cells with higher CD81 expression (transfected with pcDNA3.1-CD81) were able to bind a high density of 7P-FITC. In contrast, CD4^+^ T cells with lower CD81 expression (transfected with pcDNA3.1 only) were able to bind a low density of 7P-FITC. These results demonstrate that CD81 is significantly upregulated during Th9 cell differentiation, and 7P binds to CD81 *in vitro* (Figures 4(e) and 4(f)).

### 3.4. 7P Blocks CD81 Signaling to Inhibit Th9 Cell Differentiation *In Vitro* and *In Vivo*

**Figure 5:**
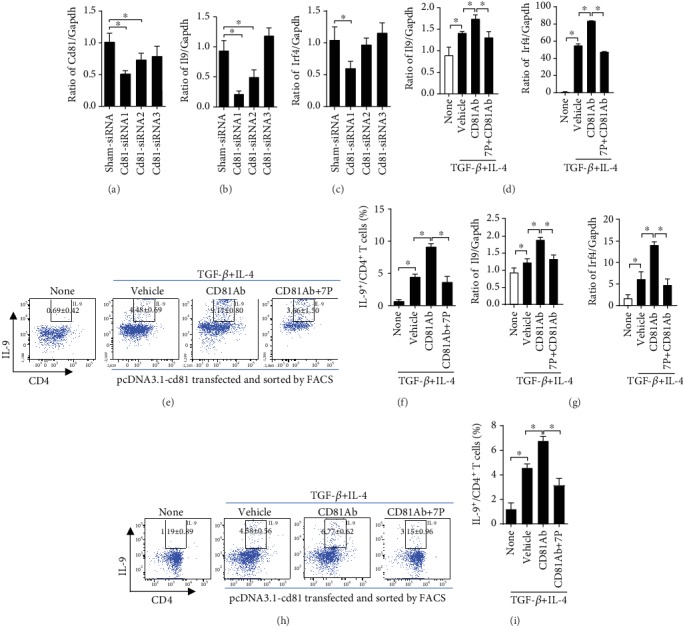
Inhibition of CD81 by siRNAs or 7P significantly reduces Th9 cell differentiation from naïve CD4^+^ T cells. (a–c) Three CD81 siRNAs were transfected into naïve CD4^+^ T cells. Mouse naïve CD4^+^ T cells transfected with CD81 siRNAs were differentiated into Th9 cells with anti-CD3 (3 *μ*g/mL), anti-CD28 (3 *μ*g/mL), anti-INF-*γ* (3 *μ*g/mL), TGF-*β* (10 ng/mL), and IL-4 (10 ng/mL) for 4–5 days. *Cd81*, *Il9*, and *Irf4* mRNA were then examined in three CD81 siRNA-transfected cells and sham siRNA-transfected cells. Data are expressed as mean ± SEM (*n* = 3 independent experiments). ^∗^*p* < 0.05 compared with sham siRNA cells. The human cDNA-CD81 plasmid was successfully transfected into primary human peripheral blood naïve CD4^+^ T cells and the Jurkat cell line. Human CD81 overexpression dramatically promotes Th9 cell differentiation *in vitro* in human primary naïve CD4^+^ T cells and the Jurkat cell line, as revealed by (d, f) qPCR and (e, g) FACS. (e, g) CD81 signaling was activated with an anti-CD81 antibody, and Th9 cell differentiation was promoted in CD81-overexpressed human primary naïve CD4^+^ T and Jurkat cells. Anti-CD81 antibody during Th9 cell differentiation is significantly inhibited by 7P in both overexpressed CD81 in human primary naïve CD4^+^ T and Jurkat cells (*n* = 5, ^∗^*p* < 0.05).

To determine the effect of 7P-CD81 signaling in regulating Th9 cell differentiation, we examined *Il9* and *Irf4* mRNA levels during Th9 cell differentiation *in vitro* after *Cd81* knockdown by siRNAs in Th9 cells. *Cd81-siRNA1* significantly inhibited Cd81 mRNA expression by *Cd81-siRNA-1* (Figure 5(a)). The expression of *Il9* and *Irf4* mRNA was markedly lower in Th9 cells differentiated from *Cd81-siRNA1*-transfected naïve CD4^+^ T cells compared to those transfected with *Sham-siRNA* and *Cd81-siRNA3* (Figures 5(b) and 5(c)). The results suggested that the CD81 signaling pathway promoted Th9 cell differentiation from naïve CD4^+^ T cells.

To further confirm the effect of 7P on CD81 signaling in Th9 differentiation, we constructed a pcDNA3.1-CD81 expression plasmid (Supplementary [Supplementary-material supplementary-material-1]). We transfected this plasmid into human Jurkat lymphocyte cells and further treated CD81-overexpressed Jurkat cells, normal Jurkat cells, human naïve CD4^+^ T cells, and human naïve CD4^+^ T cells with TGF-*β* plus IL-4- and CD81-activated antibody for 5 days. This was done with or without 7P blocking, and the development of the Th9 cell phenotype was examined. The results indicated that CD81-overexpressed cells produced higher levels of *Il9* and *Irf4* mRNA than normal Jurkat cells (Figure 5(d)) and human naïve CD4^+^ T cells (Figure 5(f)). 7P significantly blocked the *Il9* and *Irf4* mRNA transcripts promoted by anti-CD81 antibody in CD81-overexpressed human naïve CD4^+^ T cells and Jurkat cells. IL-9^+^/CD4^+^ T cell analysis via FACS also obtained results consistent with this information (Figures 5(e) and 5(g)). These data indicated that 7P blocked CD81 signaling, thus decreasing Th9 cell differentiation.

## 4. Discussion

CD81-deficient mice have been known to exhibit impaired Th2 cell responses, reduced AHR, and decreased airway inflammation. This occurs by influencing Ag-specific IgE production and regulating local cytokine production [30]. In this study, we reported that 7P blocked CD81 signaling, significantly reducing lung Th9 cell differentiation during allergic lung inflammation. Our novel findings include (1) 7P significantly suppressed allergic lung inflammation and lung Th9 cell differentiation during OVA-induced allergic lung inflammation *in vivo*; (2) 7P not only decreased Th9 cell differentiation from naïve CD4^+^ T cells but also inhibited Th9 cell development from memory CD4^+^ T cells *in vitro*; (3) 7P could bind to CD81, which was highly expressed in Th9 cells, and CD81 signaling promoted Th9 cell development *in vitro*; and (4) 7P blocked CD81 signaling to reduce Th9 cell differentiation after OVA sensitization/exposure.

Th9 cells are a unique subset of effector T cells distinct from the Th1, Th2, and Th17 cell subsets. Th9 cells secrete IL-9, IL-10, and other cytokines, which play essential roles in human asthma pathogenesis [31, 32]. In this study, we found that 7P significantly decreased allergic lung inflammation and lung Th9 cell differentiation during OVA-induced allergic lung inflammation. Based on this, we determined that 7P is a critical negative regulator of Th9 cell differentiation and IL-9 production during allergic lung inflammation. 7P significantly decreased this process, as well as suppressing lung Th9 cell differentiation during OVA-induced allergic lung inflammation *in vivo*. The results suggested that IL-9-secreting Th9 cells play a critical role in allergic lung inflammation. Therefore, inhibiting IL-9 production may be another potent avenue for treating asthma. Recently, the anti-IL-9 antibody has been used to treat asthma patients in the clinical trial (phase II) at the NIH [33, 34]. 7P could dramatically suppress Th9 cell differentiation, suggesting that 7P may be a good candidate for reducing IL-9 production in asthma.

Our findings determined that 7P bound to CD81 and blocked the CD81 receptor, inhibiting lung Th9 cell differentiation. These results present data that contradicts previous results, which have indicated that HVR1 does not bind to CD81 and that HVR2 does so [35]. As 7P is a mimic peptide derived from the HVR1 of HCV, we questioned the reason why 7P bound to CD81 on Th9 cells in our results. Although a study has reported that HVR1 could not bind to CD81, it has been demonstrated that HVR of HCV interacts with hepatocytes and CD4^+^ and CD8^+^ T cells through surface CD81 molecules [14, 15]. It was therefore difficult to determine whether HVR1 of HCV binds to CD81. Our data here provides strong evidence to support that 7P could bind to CD81 in Th9 cells. CD81 is a widely expressed cell-surface protein involved in a variety of biological responses, mostly studied in the context of the immune system [36]. CD81 is also an important factor in airway inflammation and Th9 cell differentiation during allergic lung inflammation. CD81 and CD28 co-stimulate T cells through a distinct pathway [37], and CD81 may result in the activation of TCR*γδ* T cells [38]. Human CD81 directly enhances Th1 and Th2 cell activation but preferentially induces Th2 cell proliferation upon long-term stimulation [39]. The presence of CD81 on B cells promotes IL-4 secretion and antibody production during the Th2 immune response [29]. It was reported that CD81-deficient mice had reduced airway inflammation, AHR, and Th2 cell differentiation in allergic lung inflammation [40]. CD81 expression in CD4^+^ T cells is induced by Th9-inducing factors during asthma attacks. CD81 signaling causes several proinflammatory effects in the lungs, including bronchoconstriction and eosinophilia. In contrast, 7P has a protective effect in models of allergic inflammation by suppressing bronchoconstriction and Th9 cell proliferation via blocking CD81 signaling. Previous data have shown that CD81 promotes Ag-induced lung inflammation and that 7P has a protective role in allergic lung inflammation. Consistent with these findings, we found that 7P significantly reduced Th9 cell differentiation in mice. Blocking CD81 signals leads to decreased Th9 cell differentiation and IL-9 secretion, which may then contribute to the decreased lung inflammation observed in 7P-treated mice. More importantly, this result is consistent with the previously known inhibitory role of HVR1 in IL-9 expression [12].

Our study further defined the precise molecular mechanisms underlying the regulation of Th9 cell differentiation by 7P-CD81 signaling. CD81 receptor knockdown in naïve CD4^+^ T cells by siRNA significantly decreased Th9 cell differentiation. The role of CD81 was further confirmed using CD81-overexpressing human CD4^+^ T cells. Our results suggest that 7P plays a vital role in regulating Th9 cell differentiation by altering CD81-receptor signals during allergic lung inflammation. Furthermore, we determined that 7P also controlled the differentiation of human T cells to Th9 cells. Similar to observations found in murine studies, 7P diminished the differentiation of human naïve CD4^+^ T cells in peripheral blood to Th9 cells, further indicating that 7P may be a good candidate for reducing IL-9 production in asthma.

## 5. Conclusions

We have provided *in vitro* and *in vivo* evidence to demonstrate that 7P inhibits Th9 cell differentiation and allergic lung inflammation by blocking CD81 signaling, thus downregulating Th9 differentiation. Our results demonstrate the important role of 7P-CD81 signaling in regulating Th9 cell differentiation.

## Data Availability

The data used to support the findings of this study are available from the corresponding author upon request.

## Supplementary Materials

Supplementary Materials Supplemental Figure 1: 7P significantly decreased eosinophil number of BALF and mast cell number of allergic lungs during OVA-induced allergic lung inflammation. (A) The cell differential analysis of BALF after 7P administration (*n* = 5, ^∗^*p* < 0.05); (B) 7P dramatically decreased eosinophil number in BALF (*n* = 5, ^∗^*p* < 0.05); (C) frozen lung tissue sections treated with none, vehicle, sham-7P, and 7P were stained with PAS staining; (D) PAS-positive cells were quantified with a confocal field (at ×40) (*n* = 3, *p* < 0.05). Supplemental Figure 2: 7P dramatically inhibits BALF, blood, lymph nodes, and spleen Th9 cell differentiation during allergic lung inflammation after OVA sensitization and exposure in vivo. (A) C57BL6J mice (*n* = 8 each) were sensitized with OVA in adjuvant. 14 to 21 days later, mice were exposed to inhale OVA for four consecutive days. Mice were implanted minipumps from day 13 to day 19; 7P and sham-7P were given 6 *μ*g/mouse. The percentages of IL-9^+^CD4^+^ T cells in BALF, blood, lymph nodes, and spleen from 7P versus sham-7P and vehicle minipumped mice were analyzed by flow cytometry 48 hours after the last OVA exposure (^∗^*p* < 0.05, *n* = 12); IL-9, IL-5, and IL-13 protein levels in BALF were quantified by ELISA (^∗^*p* < 0.05). Supplemental Figure 3: pcDNA3.1-CD81 plasmid was constructed, and CD18 expression was analyzed by flow cytometry. (A) Human CD81 was cloned into a pcDNA3.1 vector with EcoRI and BamHI digesting sites to construct a highly expressed human CD81 plasmid. (B) T cells transfected with pcDNA3.1-CD81 were examined by flow cytometry with anti-human CD81 antibody.
