# Supplementary material for: A 7-Amino Acid Peptide Mimic from Hepatitis C Virus Hypervariable Region 1 Inhibits Mouse Lung Th9 Cell Differentiation by Blocking CD81 Signaling during Allergic Lung Inflammation
Source: J Immunol Res. 2020 Mar 20;2020:4184380. doi: 10.1155/2020/4184380 (PMC7109583; doi:10.1155/2020/4184380)
Supplement: Supplementary Materials — Supplemental Figure 1: 7P significantly decreased eosinophil number of BALF and mast cell number of allergic lungs during OVA-induced allergic lung inflammation. (A) The cell differential analysis of BALF after 7P administration (n = 5, ∗p < 0.05); (B) 7P dramatically decreased eosinophil number in BALF (n = 5, ∗p < 0.05); (C) frozen lung tissue sections treated with none, vehicle, sham-7P, and 7P were stained with PAS staining; (D) PAS-positive cells were quantified with a confocal field (at ×40) (n = 3, p < 0.05). Supplemental Figure 2: 7P dramatically inhibits BALF, blood, lymph nodes, and spleen Th9 cell differentiation during allergic lung inflammation after OVA sensitization and exposure in vivo. (A) C57BL6J mice (n = 8 each) were sensitized with OVA in adjuvant. 14 to 21 days later, mice were exposed to inhale OVA for four consecutive days. Mice were implanted minipumps from day 13 to day 19; 7P and sham-7P were given 6 μg/mouse. The percentages of IL-9+CD4+ T cells in BALF, blood, lymph nodes, and spleen from 7P versus sham-7P and vehicle minipumped mice were analyzed by flow cytometry 48 hours after the last OVA exposure (∗p < 0.05, n = 12); IL-9, IL-5, and IL-13 protein levels in BALF were quantified by ELISA (∗p < 0.05). Supplemental Figure 3: pcDNA3.1-CD81 plasmid was constructed, and CD18 expression was analyzed by flow cytometry. (A) Human CD81 was cloned into a pcDNA3.1 vector with EcoRI and BamHI digesting sites to construct a highly expressed human CD81 plasmid. (B) T cells transfected with pcDNA3.1-CD81 were examined by flow cytometry with anti-human CD81 antibody. [file 4184380.f1.docx]

**Supplemental figures**


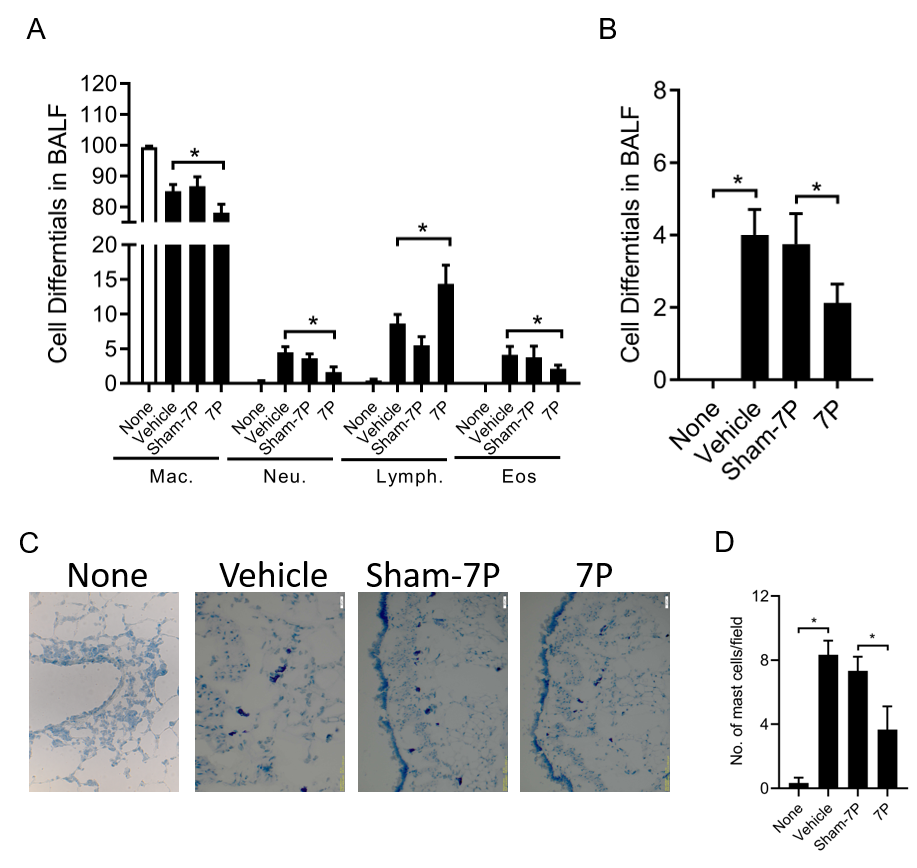


**Supplemental Figure 1**. 7P significantly decreased eosinophils of BALF and mast cell number of allergic lungs during OVA-induced allergic lung inflammation. (A) the cell differential analysis of BALF after 7P administration (n=5, *p<0.05); (B) 7P dramatically decreased eosinophil number in BALF (n=5, *p<0.05); (C) Frozen lung tissue sections treated with none, vehicle, Sham-7P and 7P were stained with PAS staining, (C) PAS positive cells were quantified with a confocal field (at x40 ) (n=3, p<0.05).


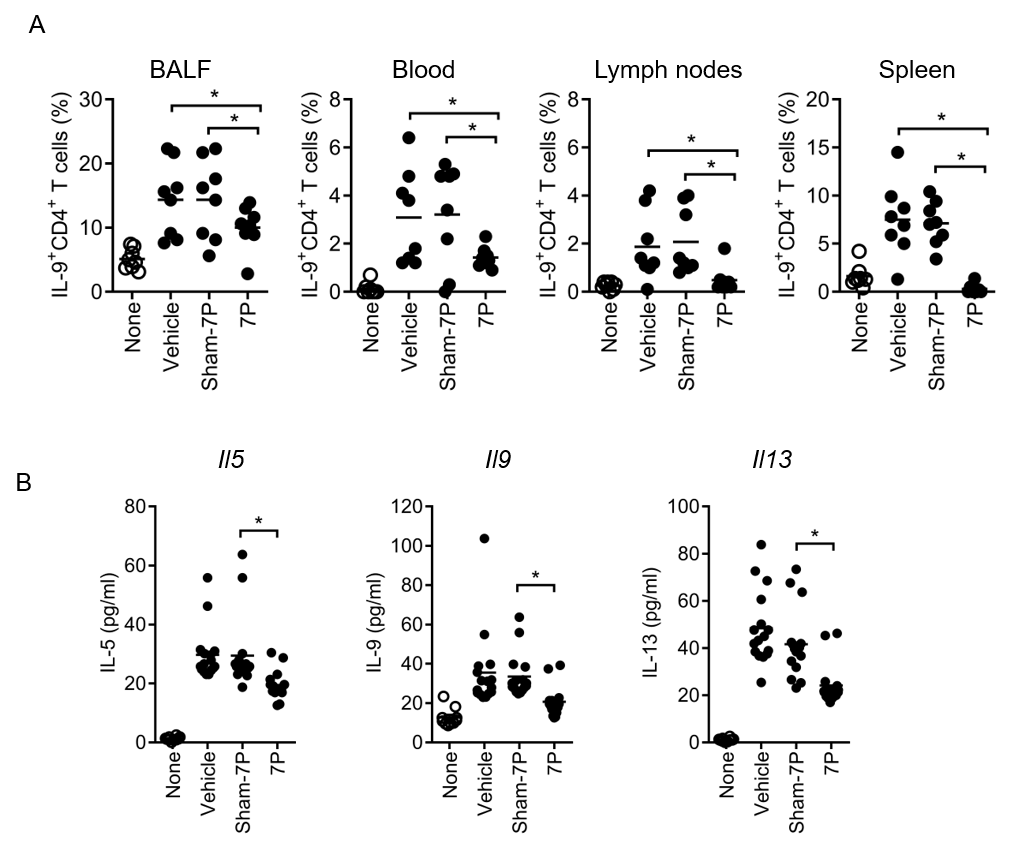


**Supplemental Figure 2.** 7P dramatically inhibits BALF, blood, lymph nodes and spleen Th9 cell differentiation during allergic lung inflammation after OVA sensitization and exposure *in vivo*. (A) C57BL6J mice (n = 8 each) were sensitized with OVA in adjuvant. 14 to 21 days later, mice were exposed to inhale OVA for four consecutive days. Mice were implanted mini-pumps from day 13 to day 19; 7P and sham-7P were given 6 μg/mouse. The percentages of IL-9^+^CD4^+^ T cells in BALF, blood, lymph nodes and spleen from 7P *versus* sham-7P and vehicle mini-pumped mice were analyzed by flow cytometry 48 hours after the last OVA exposure ( *p<0.05, n=12); IL-9, IL-5 and IL-13 protein levels in BALF were quantified by ELISA. (*p<0.05).


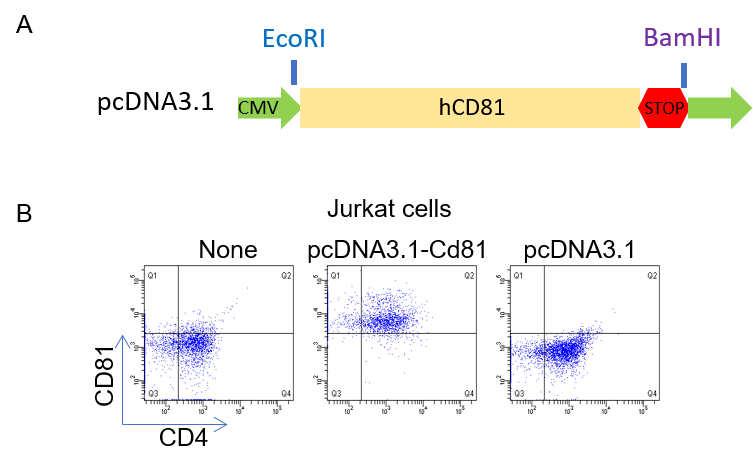


**Supplemental Figure 3:** pcDNA3.1-CD81 plasmid was constructed and CD18 expression was analyzed by flow cytometry. (A) Human CD81 was cloned into a pcDNA3.1 vector with EcoRI and BamHI digesting sites to construct a highly expressed human CD81 plasmid. (B) CD81^+^ T cells transfected with pcDNA3.1-CD81 were examined by flow cytometry.
